# Disentangling causal relationships linking the oral microbiome, metabolism, inflammation, and dental caries via Mendelian randomization

**DOI:** 10.1097/MD.0000000000048263

**Published:** 2026-04-24

**Authors:** Qiaomei Liang, Tingyan Zeng

**Affiliations:** a Department of Stomatology, The Second Hospital of Hunan University of Chinese Medicine, Changsha, China; b Department of Stomatology, The Third Hospital of Changsha (The Affiliated Changsha Hospital of Hunan University), Changsha, China.

**Keywords:** blood metabolome, dental caries, inflammatory proteins, Mendelian randomization, oral microbiota

## Abstract

Dental caries is a major global health burden. While observational studies suggest links between the oral microbiome, metabolism, inflammation, and caries, causal relationships remain unclear due to confounding and reverse causation. This study aimed to systematically dissect both the causal roles and the interplay between these factors in caries etiology. We employed a two-sample Mendelian randomization (MR) framework using large-scale genome-wide association study summary statistics. Univariable MR was used to assess the direct causal effects of oral microbial taxa, circulating metabolites, and inflammatory proteins on caries risk. Multivariable MR and 2-step MR were subsequently applied to perform mediation analysis and disentangle complex causal pathways. Effect sizes are reported as odds ratios (ORs) with 95% confidence intervals (CIs). Univariable MR identified protective causal effects of the genera *Haemophilus* (OR = 0.965, 95% CI: 0.937–0.994) and *Rothia* (OR = 0.965, 95% CI: 0.934–0.996) on caries risk. Genetically predicted higher levels of N4-acetylcytidine and pyrraline were associated with increased risk, whereas eicosapentaenoate showed a protective effect. The inflammatory proteins C-X-C motif chemokine 11 and signaling lymphocytic activation molecule family member 1 were causally associated with higher caries risk. Crucially, mediation analysis revealed that the protective effect of *Haemophilus* was partly mediated through its influence on circulating gamma-glutamylthreonine and X-11483 (an untargeted metabolomics feature ID). Our findings provide robust causal evidence for an integrated oral microbiome–metabolism–inflammation axis in caries etiology. These results highlight novel biomarkers for risk stratification and potential therapeutic targets, offering a scientific basis for developing more effective preventive strategies against this prevalent disease.

## 1. Introduction

Dental caries represents one of the most prevalent and burdensome oral diseases globally, substantially impairing patients’ quality of life and imposing significant health and economic burdens.^[1,2]^ Its etiology is multifactorial: high-frequency sugar intake creates an acidic milieu that, together with host and environmental factors, drives biofilm dysbiosis and the enrichment of acidogenic taxa.^[3]^ Beyond local mechanisms, converging multi-omics evidence indicates that caries is intimately linked to host metabolic and inflammatory networks. For instance, elevated inflammatory markers and altered metabolite profiles are correlated with disease severity.^[4–7]^ While bidirectional crosstalk between chronic inflammation and dysbiosis has been reported along the oral–gut–liver axis,^[8,9]^ current evidence derives largely from observational studies, which are insufficient to establish directional causality.

Although observational studies have consistently associated microbial community shifts and metabolic profiles with caries,^[10–12]^ these findings remain vulnerable to residual confounding, reverse causation, and temporal ambiguity. Even sophisticated adjustment models often fail to eliminate systematic bias.^[13–15]^ To address these limitations, Mendelian randomization (MR) utilizes genetic variants as instrumental variables (IVs) to provide quasi-experimental evidence for causality.^[16,17]^ However, prior MR studies have predominantly assessed single exposures in isolation,^[18,19]^ limiting the dissection of independent effects and mediation pathways. Multivariable MR (MVMR) overcomes this by enabling simultaneous assessment of correlated exposures, quantifying each one’s independent effect after mutual adjustment, and allowing rigorous mediation analysis to dissect mechanistic pathways – thereby disentangling the oral microbiome–metabolism–inflammation axis in caries etiology.

We hypothesize that oral dysbiosis, circulating metabolic dysregulation, and inflammatory mediators operate as interconnected causal risk factors for caries. Accordingly, this study employed large-scale genome-wide association study (GWAS) summary statistics within a two-sample MR framework to estimate the direct causal effects of oral microbiome taxa, circulating metabolites, and inflammatory proteins on caries risk via univariable MR (UVMR); quantify the independent effect of each exposure after mutual adjustment using MVMR, thereby probing potential interactions by correlated factors; and dissect potential mechanistic pathways through mediation analysis. Collectively, this work aims to provide robust causal evidence to inform the identification of early-warning biomarkers and therapeutic targets for caries.

## 2. Methods

### 2.1. Overall framework and analytical workflow

**Figure 1.**
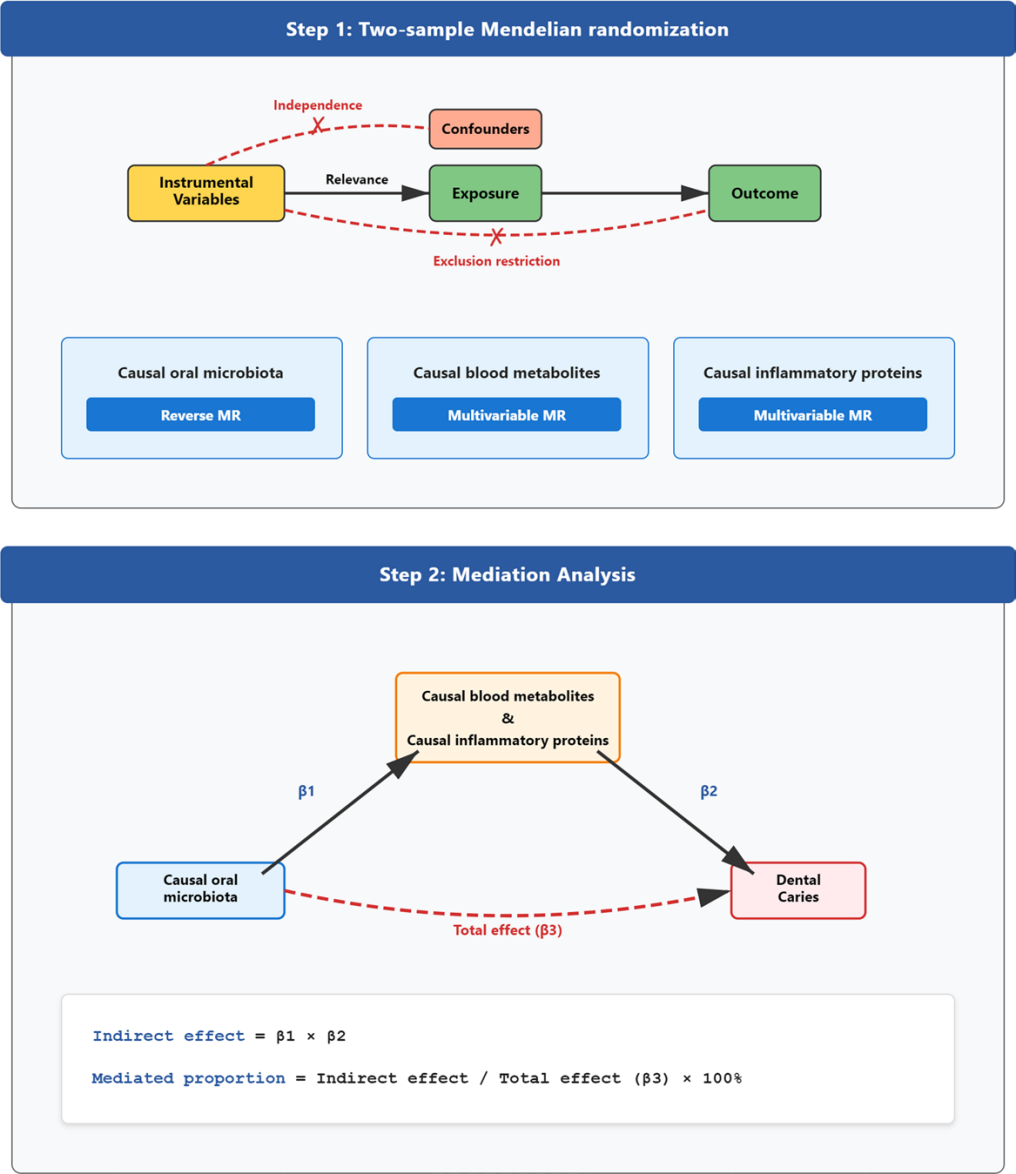
Study design for Mendelian randomization analysis of oral microbiota, blood metabolites, inflammatory proteins, and dental caries. This figure illustrates the two-sample MR and mediation MR framework. Analysis steps: two-sample MR to assess causality and mediation MR to evaluate blood metabolites and inflammatory proteins as mediators. MR = Mendelian randomization.

We systematically investigated the oral microbiome’s role in caries development and progression and evaluated potential mediation by circulating metabolites and inflammatory proteins. Figure 1 delineates this analytical workflow. We built a comprehensive MR framework using GWAS summary data. The workflow started with UVMR to estimate the direct causal effect of oral microbiome taxa on caries, using curated GWAS summary statistics (Section 2.3) and rigorously selected IVs (Section 2.4). To evaluate reverse causality, we also conducted reverse-direction UVMR. We then applied UVMR to test the effects of oral microbiome taxa on circulating metabolites/inflammatory proteins, followed by UVMR to test whether these circulating traits causally influence caries risk, thereby nominating putative mediators that satisfy both criteria (Section 2.5). Finally, we used MVMR (Section 2.6) to delineate and quantify the independent roles of candidate mediators in the microbiome-to-caries pathway and to estimate indirect effects. We implemented a prespecified suite of sensitivity analyses (Section 2.7) to assess core MR assumptions and enhance robustness. This study adheres to STROBE-MR reporting guidelines (Table S1, Supplemental Digital Content, https://links.lww.com/MD/R705).^[20]^

### 2.2. MR principles and core assumptions

Traditional observational studies of associations between the oral microbiome and complex diseases such as caries are susceptible to confounding and reverse causation. MR uses genetic variants as IVs to obtain less biased causal estimates by capitalizing on the random allocation of alleles at conception. The validity of MR relies on 3 core assumptions: relevance, IVs are strongly associated with the exposure; independence, IVs are independent of confounders of the exposure–outcome relationship; and exclusion restriction, IVs affect the outcome solely via the exposure, not through alternative pathways. Our design and sensitivity analyses were tailored to evaluate these assumptions.^[21]^

### 2.3. Data sources and description

We integrated 4 publicly available large-scale GWAS summary datasets covering exposures (oral microbiome), putative mediators (circulating metabolites and inflammatory proteins), and the outcome (caries). Oral microbiome GWAS summary statistics came from 610 Danish participants,^[22]^ with genetic associations for 43 oral microbial taxa. Caries GWAS data came from the FinnGen R11 database, which comprised 9446 cases and 288,472 controls. Metabolomics GWAS summary statistics were obtained from the Canadian Longitudinal Study on Aging,^[23]^ including 51,338 individuals and approximately 1400 metabolites spanning amino acids, carbohydrates, lipids, nucleotides, and related classes. GWAS data for 91 inflammatory proteins were drawn from 14,824 individuals of European ancestry.^[24]^ Participants were predominantly of European descent, and sample overlap was minimal, supporting the sample independence assumption in two-sample MR.

### 2.4. Selection and validation of genetic IVs

To ensure IV validity, we adopted stringent selection criteria. First, acknowledging limited sample sizes for some GWAS, we used a suggestive significance threshold of *P* < 5 × 10^−5^ for initial single-nucleotide polymorphism (SNP) screening to balance IV strength and instrument count for adequate power.^[19]^ Second, to reduce linkage disequilibrium (LD) between IVs, we applied LD clumping using the 1000 Genomes Project Phase 3 European reference panel with a 10,000-kb window and *r*^2^ = 0.001, ensuring approximate independence. Third, to mitigate weak-instrument bias, we calculated the *F*-statistic for each SNP (*F* = [β/SE]^2^) and retained only SNPs with *F* ≥ 10.^[18]^

### 2.5. UVMR analysis

We implemented UVMR to estimate the causal effect of each exposure on the outcome using the selected IVs. To address horizontal pleiotropy, we employed 3 complementary estimators via the TwoSampleMR R package (version 0.6.7; MRCIEU, University of Bristol, Bristol, United Kingdom, https://github.com/MRCIEU/TwoSampleMR): inverse-variance weighted (primary analysis),^[25]^ which is efficient under no or balanced pleiotropy; weighted median,^[26]^ which remains consistent when up to 50% of IVs are invalid; and Mendelian randomization egger regression (MR-Egger),^[27]^ which tests and adjusts for directional pleiotropy at the cost of power. We triangulated across these estimators to assess robustness. Circulating metabolites or inflammatory proteins demonstrating significant causal associations with caries were designated as candidate mediators for downstream analyses. To qualify as a potential mediator, a circulating metabolite or inflammatory protein had to satisfy 2 sequential criteria, first demonstrating significant causal association with the exposure where oral microbiome taxa influenced the mediator (*P* < .05), and second showing significant causal association with the outcome where the mediator influenced caries risk (*P* < .05), with directional consistency maintained across the complete mediation pathway. Candidate mediators meeting these initial screening criteria were then advanced to multivariable analysis for further evaluation of their independent causal roles.

### 2.6. MVMR and mediation analysis

Using the candidate mediators identified in Section 2.5, we applied MVMR to estimate the direct effects of multiple exposures (e.g., 1 microbial taxon and 1 mediator) on caries while accounting for genetic correlations among exposures.^[28]^ MVMR thereby separates the direct effect of the microbial taxon (independent of the mediator) from the mediator’s effect on caries (adjusted for the taxon). Only mediators retaining statistical significance (*P* < .05) in MVMR were considered as confirmed mediators for downstream mediation quantification. We quantified the indirect effect via the product-of-coefficients method, multiplying the microbiota-to-mediator effect from UVMR (β_1_) by the mediator-to-caries effect from MVMR (β_2_).^[29]^ The proportion mediated was (β_1_ × β_2_)/β_3_, where β_3_ is the total microbiota-to-caries effect from UVMR. MVMR thus offers a deeper level of causal inference for dissecting potential mechanistic pathways and assessing the importance of identified mediators.

### 2.7. Heterogeneity, pleiotropy, and sensitivity analyses

We used Cochran *Q* to assess heterogeneity among IVs (*P* < .05, suggesting heterogeneity). To evaluate horizontal pleiotropy, we used the MR-Egger intercept (*P* < .05, indicating directional pleiotropy) and the Mendelian randomization pleiotropy residual sum and outlier (MR-PRESSO) global test, which detects outliers and provides outlier-corrected estimates (*P* < .05, indicating significant pleiotropy). When MR-PRESSO identified outliers, we repeated analyses after their removal. Collectively, these sensitivity analyses provided multiple layers of validation for MR assumptions and enhanced the credibility of our findings.

## 3. Results

### 3.1. Causal effects of the oral microbiome on caries

**Figure 2.**
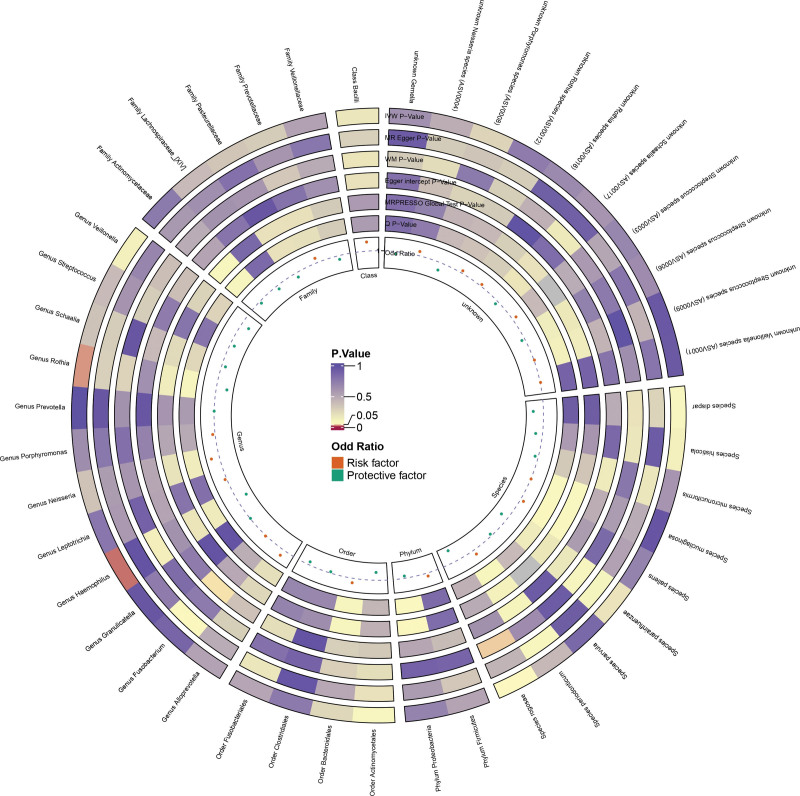
Causal associations between oral microbiota and dental caries using two-sample Mendelian randomization. Odds ratios (ORs) and 95% confidence intervals (CIs) were estimated using the inverse-variance weighted (IVW) method, with sensitivity analyses via MR-Egger and weighted median methods. MR-Egger = Mendelian randomization egger regression, MR-PRESSO = Mendelian randomization pleiotropy residual sum and outlier, WM = weighted median.

To test whether oral dysbiosis is etiologic for caries, we conducted UVMR for multiple oral microbial taxa. The analysis identified 2 taxa, genus *Haemophilus* (odds ratio [OR] = 0.965, *P* = .018) and genus *Rothia* (OR = 0.965, *P* = .027), that exhibited statistically significant protective effects against caries (Fig. 2). Full results and sensitivity analyses are detailed in Tables S2 and S3, Supplemental Digital Content, https://links.lww.com/MD/R705. Notably, weighted median and MR-Egger estimates were directionally consistent, and neither MR-PRESSO nor the MR-Egger intercept indicated evidence of outlier-driven or directional pleiotropy. Reverse-direction MR further ruled out reverse causation (Table S4, Supplemental Digital Content, https://links.lww.com/MD/R705). Collectively, these findings support robust protective causal associations for both *Haemophilus* and *Rothia*.

### 3.2. Causal effects of circulating metabolites on caries

**Figure 3.**
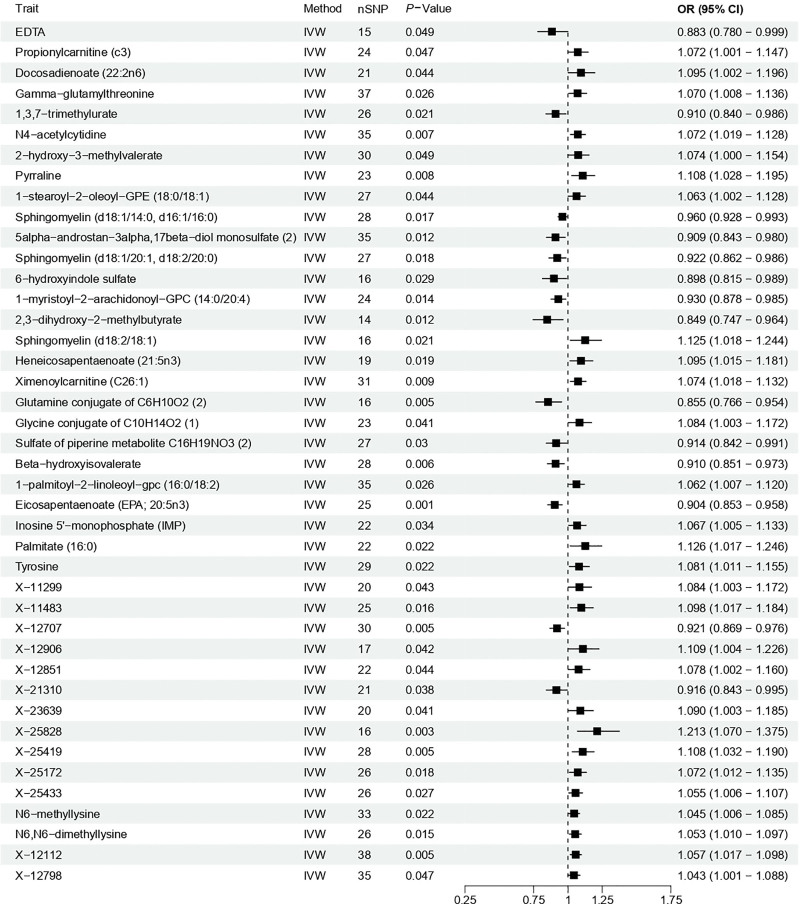
Causal associations between blood metabolites and dental caries using two-sample Mendelian randomization. The inverse-variance weighted method was primary, supported by MR-Egger and weighted median methods. Significance threshold: *P* *<* .05. CI = confidence interval, EDTA = ethylenediaminetetraacetic acid, IVW = inverse-variance weighted, MR-Egger = Mendelian randomization egger regression, OR = odds ratio, SNP = single-nucleotide polymorphism.

We further evaluated causal associations between circulating metabolites and caries. Inverse-variance weighted analysis identified 42 metabolites spanning diverse biochemical classes with statistically significant associations. Among these, the most prominent risk-increasing associations were observed for genetically higher N4-acetylcytidine (ac4C; OR = 1.072, *P* = .007) and pyrraline (OR = 1.108, *P* = .008). Conversely, the strongest protective associations were identified for higher eicosapentaenoate (EPA; OR = 0.904, *P* = .0006) and a glutamine conjugate of C_6_H_10_O_2_ (2) (OR = 0.855, *P* = .005; Fig. 3). The complete results encompassing all tested metabolites are provided in Table S5, Supplemental Digital Content, https://links.lww.com/MD/R705. Additionally, sensitivity analyses largely corroborated these findings, with no evidence of significant pleiotropy or influential outliers (Table S6, Supplemental Digital Content, https://links.lww.com/MD/R705). These metabolites may thus represent candidate biomarkers or therapeutic targets.

### 3.3. Causal effects of inflammatory proteins on caries

**Figure 4.**
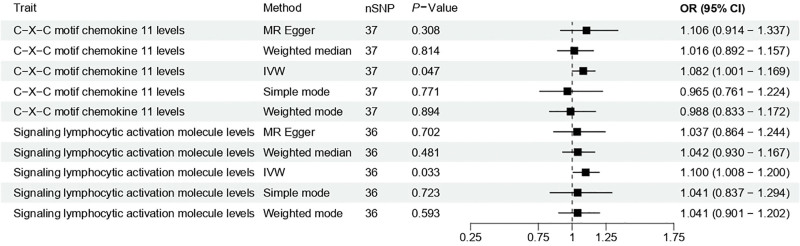
Causal associations between inflammatory proteins and dental caries using two-sample Mendelian randomization. The inverse-variance weighted method was used, with MR-Egger and weighted median validation. Significance threshold: *P* *<* .05. CI = confidence interval, IVW = inverse-variance weighted, MR-Egger = Mendelian randomization egger regression, OR = odds ratio, SNP = single-nucleotide polymorphism.

We next explored the potential causal associations between inflammatory proteins and caries risk. Among the 91 proteins evaluated, 2 showed statistically significant causal relationships. Genetically predicted higher levels of C-X-C motif chemokine ligand (CXCL) 11 (OR = 1.082, *P* = .047) and signaling lymphocytic activation molecule family member 1 (SLAMF1; OR = 1.099, *P* = .033) were both associated with an increased caries risk (Fig. 4). Complete results for all 91 tested inflammatory proteins are presented in Table S7, Supplemental Digital Content, https://links.lww.com/MD/R705. Consistency across sensitivity analyses further supported the reliability of these associations (Table S8, Supplemental Digital Content, https://links.lww.com/MD/R705).

### 3.4. Mediation analysis

**Figure 5.**
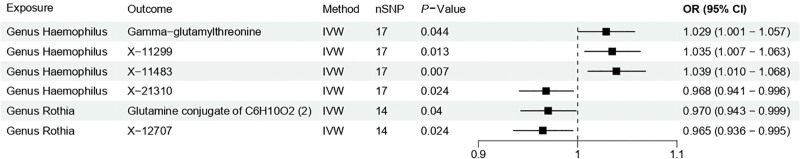
Causal associations between oral microbiota and blood metabolites using two-sample Mendelian randomization. The inverse-variance weighted method was used, with MR-Egger and weighted median validation. Significance threshold: *P* *<* .05. CI = confidence interval, IVW = inverse-variance weighted, MR-Egger = Mendelian randomization egger regression, OR = odds ratio, SNP = single-nucleotide polymorphism.

To identify potential mechanistic pathways, we performed a 2-step MR to test whether circulating metabolites mediate the protective effects of oral microbiota on caries risk. First, UVMR assessed the causal effects of *Haemophilus* and *Rothia* on circulating metabolites. *Haemophilus* demonstrated positive associations with gamma-glutamylthreonine (OR = 1.029, *P* = .044) and X-11483 (OR = 1.039, *P* = .007), whereas *Rothia* showed negative causal associations with a glutamine conjugate of C_6_H_10_O_2_ (2) (OR = 0.970, *P* = .040) and X-12707 (OR = 0.965, *P* = .024; Fig. 5). Complete results for all 42 tested metabolites are presented in Table S9, Supplemental Digital Content, https://links.lww.com/MD/R705. No inflammatory proteins were identified as significant mediators of the microbiota–caries pathway. Subsequent MVMR analysis demonstrated that, after adjusting for *Haemophilus*, both gamma-glutamylthreonine (OR = 0.964, *P* = .009) and X-11483 (OR = 0.967, *P* = .020) retained statistically significant protective associations with caries (Table 1). This finding suggests that *Haemophilus* may exert part of its protective effect by modulating these metabolites.

**Table 1:** Mediation analysis of oral microbiota effects on dental caries via blood metabolites.

Exposure	Adjusted factors	Inverse-variance weighted		
		OR	95% CI	*P* value
Genus *Haemophilus*	Gamma-glutamylthreonine	0.964	0.939–0.991	.009
Gamma-glutamylthreonine	Genus *Haemophilus*	1.129	1.014–1.257	.026
Genus *Haemophilus*	X-11483	0.967	0.940–0.995	.020
X-11483	Genus *Haemophilus*	1.134	1.004–1.280	.043

CI = confidence interval, OR = odds ratio.

## 4. Discussion

Multi-omics evidence indicates that integrating the oral microbiome with metabolomics is essential to reveal the microbiome’s functional contributions to caries and its pathophysiology.^[30]^ In addition, inflammatory and immune mediators also shape the progression and activity of caries. At the same time, compared with observational studies, MR reduces confounding and reverse causation, enabling stronger causal tests of oral exposures and their effects on caries.^[31]^ Within this context, we used UVMR/MVMR to interrogate the oral microbiome–circulating metabolites/inflammatory proteins–caries axis and to explore metabolic mediation through 2-step MR. Our genetic evidence suggests protective associations between *Haemophilus* and *Rothia* with reduced caries risk. We identified multiple circulating metabolites with directionally consistent effects on caries risk, with ac4C and pyrraline associated with increased risk and EPA and a glutamine conjugate of C_6_H_10_O_2_ (2) associated with decreased risk. At the inflammatory-protein level, genetically higher CXCL11 and SLAMF1 were associated with increased caries risk. Moreover, MVMR supported a protective mediation pathway through *Haemophilus*–gamma-glutamylthreonine/X-11483-caries, thereby highlighting genetically supported mechanistic pathways across the microbiome–metabolism–caries axis.

As key members of the oral symbiotic community, *Haemophilus* is associated with oral health. Functional genomic studies indicate that it helps maintain a favorable host–microbe ecology. Population studies report higher *Haemophilus* abundance in health-associated, low-cariogenic individuals,^[11]^ and functional evidence shows that *Haemophilus parainfluenzae* colonizes multiple oral niches with genes enabling stable biofilm growth under diverse conditions,^[32]^ providing a biological basis for its role in maintaining ecological balance against acidogenic dysbiosis. Similarly, *Rothia*, a nitrate-reducing genus, is commonly found in individuals without oral diseases. Experimental evidence demonstrates its capacity to enhance salivary antioxidant capacity, thereby contributing to caries prevention.^[33,34]^ Overall, evidence from populations and mechanisms corroborates roles for *Haemophilus* and *Rothia* in health-associated biofilms and anti-acidification ecology, aligning with our protective genetic findings and providing ecological and biological plausibility for the observed metabolic and inflammatory pathways.

Our identification of ac4C and pyrraline as risk-increasing metabolites is consistent with their roles in inflammatory pathways. As a modified RNA nucleoside, ac4C is linked to inflammatory responses. The ac4C-catalyzing enzyme N-acetyltransferase 10 has been shown to enhance macrophage inflammation via NOX2-ROS-NF-κB signaling.^[35]^ Pyrraline, as an advanced glycation end product, reflects the inflammatory burden associated with oral pathology.^[36]^ These findings suggest that circulating inflammatory metabolites may contribute to caries susceptibility. Conversely, n−3 fatty acids such as EPA inhibit multiple inflammatory pathways and serve as precursors to specialized pro-resolving mediators, including resolvins, protectins, and maresins.^[37]^ Concurrently, amino acid metabolism within oral biofilm, particularly the arginine deiminase system (ADS), enhances alkali production that neutralizes acid and reduces biofilm cariogenicity; healthy plaque exhibits higher ADS activity, and meta-analyses indicate that salivary/plaque ADS activity may serve as a caries risk indicator.^[38]^ Consistent with these mechanisms, our findings reveal that EPA and amino acid-related metabolites, including gamma-glutamyl dipeptides, have protective associations with caries, suggesting that composite pathways involving both anti-inflammatory and anti-acidification mechanisms may underpin their protective effects.

Caries progression involves not only demineralization-remineralization imbalance but also local inflammation and immune mediators.^[39]^ CXCL11, a key ligand for C-X-C chemokine receptor type 3, forms a Th1-type chemoattractant axis with CXCL9/10 that regulates immune cell migration and is established to participate in interferon-gamma-induced inflammatory amplification.^[40,41]^ SLAMF1, an immune co-receptor, positively regulates toll-like receptor 4 downstream signaling and amplifies pro-inflammatory mediator release. Mechanistic studies confirm that disrupting SLAMF1-TRIF-related adaptor molecule interactions significantly inhibits expression of interferon-beta, tumor necrosis factor, interleukin-1 beta, and interleukin-6.^[42]^ In inflammatory diseases such as NASH, elevated SLAMF1 levels reflect inflammatory activity,^[43]^ and large-scale proteomics studies show that inflammatory proteins, including CXCL11 and SLAMF1, can be elevated in circulation before clinical disease manifestation.^[44]^ In line with this evidence, our findings identify associations between genetically higher CXCL11 and SLAMF1 and increased caries risk, suggesting that the Th1 chemoattractant axis and innate immune amplification may jointly contribute to a pro-cariogenic inflammatory microenvironment.

The identification of a mediation pathway through gamma-glutamylthreonine and X-11483 represents a key finding of this study. MVMR mediation analysis demonstrated that after adjusting for *Haemophilus*, both metabolites retained significant protective associations with caries, indicating that *Haemophilus* may exert part of its effect by modulating these circulating metabolites. Biologically, gamma-glutamyl amino acids participate in the gamma-glutamyl cycle involved in amino acid transport and antioxidant metabolism.^[45]^ Notably, oral bacteria such as *Lactobacillus reuteri* have been documented to synthesize gamma-glutamyl dipeptides,^[46]^ providing a mechanism by which commensal oral microbiota may shape circulating metabolite profiles. Multi-omics studies demonstrate coordinated shifts between oral microbes and salivary/plasma metabolites, consistent with microbe-metabolite crosstalk.^[10,47]^ This genetically delineated pathway linking beneficial oral bacteria to protective metabolites and ultimately to reduced caries risk highlights the value of integrating microbiome and metabolomics data to dissect complex disease etiology and may inform future biomarker development for caries risk stratification.

Although this study provides novel insights into the oral microbiome–circulating metabolite/inflammatory protein–caries axis, several limitations must be acknowledged. First, methodologically, MR relies on key assumptions (relevance, independence, and exclusion restriction). While we employed multiple sensitivity analyses, the potential for residual horizontal pleiotropy or weak-instrument bias cannot be entirely excluded. Second, we did not apply Bonferroni or false discovery rate adjustment across our primary UVMR analyses. Given the exploratory nature of our mediation analysis, nominal *P* values were retained to maximize discovery capacity. However, this increases the risk of false-positive associations and may inflate effect estimates. Replication in independent cohorts is essential before translating findings to clinical practice. Third, we acknowledge that the *P* < 5 × 10^−5^ threshold for initial SNP screening, while justified by limited GWAS sample sizes for some exposures, may introduce weak-instrument bias compared with the genome-wide standard (*P* < 5 × 10^−8^). *F*-statistic filtering and sensitivity analyses were employed to mitigate this risk, but residual bias remains possible. Fourth, our study focused on circulating metabolites, which may not perfectly reflect the local metabolic space of saliva or plaque. Fifth, our findings are based on data from individuals of predominantly European ancestry. Due to differences in LD structure, these results require validation in diverse populations. Finally, regarding mechanism, the *Haemophilus*–gamma-glutamyl dipeptide–caries pathway, while genetically supported, requires functional validation.

Several avenues warrant future investigation. First, functional validation studies should examine whether identified protective metabolites directly influence caries-related oral biofilm properties, including acid production and demineralization potential. Second, measuring these metabolites in local oral fluids rather than circulating blood, combined with concurrent microbiota profiling, would strengthen mechanistic inference. Third, replication in populations of non-European ancestry is essential to determine whether these associations generalize across genetic backgrounds and disease prevalence patterns. Fourth, longitudinal cohort studies incorporating these genetic and metabolic markers could prospectively evaluate whether such biomarker panels predict caries incidence and progression, supporting development of clinical risk stratification tools. Finally, experimental modulation of identified protective taxa through probiotic or dietary interventions, or direct supplementation of protective metabolites, warrants investigation as potential therapeutic or preventive strategies for caries.

In conclusion, our findings suggest protective roles for commensal genera such as *Haemophilus* and *Rothia*, while genetically predicted higher levels of circulating metabolites (ac4C, pyrraline) and inflammatory proteins (CXCL11, SLAMF1) were associated with increased risk. Crucially, mediation analysis identified a potential pathway where the protective effect of *Haemophilus* is partly channeled through gamma-glutamylthreonine and X-11483. By leveraging genetic instruments, this approach strengthens causal inference beyond traditional observational studies, offering a clearer view of the molecular drivers of caries. These results highlight novel biomarkers for risk stratification and potential therapeutic targets for precision prevention. However, given the exploratory nature of this analysis, these associations require replication in independent, diverse cohorts before clinical application. Further functional studies are essential to validate the identified mechanistic pathways and translate these genetic insights into effective, personalized preventive strategies against this globally prevalent disease.

## Acknowledgments

We gratefully acknowledge the original authors for providing the datasets used in this research.

## Author contributions

**Conceptualization:** Qiaomei Liang, Tingyan Zeng.

**Data curation:** Tingyan Zeng.

**Writing – original draft:** Qiaomei Liang, Tingyan Zeng.

**Writing – review & editing:** Qiaomei Liang, Tingyan Zeng.

## Supplementary Material


